# Mechanisms linking affective reactions to competition-related and competition-extraneous concerns in male martial artists

**DOI:** 10.1111/j.1600-0838.2009.01072.x

**Published:** 2011

**Authors:** E Cerin, A Barnett

**Affiliations:** 1 Institute of Human Performance, The University of Hong KongHong Kong SAR, China; 2 USDA/ARS Children's Nutrition Research Center, Baylor College of MedicineHouston, Texas, USA; 3 School of Health and Human Performance, Central Queensland UniversityQueensland, Australia; 4 Centre of Excellence for Applied Sport Science Research, Queensland Academy of SportQueensland, Australia

**Keywords:** ESM, martial arts, emotions

## Abstract

The main aim of this study was to examine affective linkages between competition-related and competition-extraneous concern domains. A secondary purpose was to establish the contributions of pre-competition affects to post-competition performance appraisals, independent of pre-competition performance expectations. Thirty-nine highly skilled male martial artists were assessed at five random times a day for a week and 1 h before a major competition on affective states and sources of concern. They also reported their performance expectations and post-competition performance appraisals. Affective states triggered by competition-related and competition-extraneous concerns persisted in time. Carry-over effects were stronger after reports of competition-related concerns, emphasizing the subjective importance of the competitive event. Although positive (enjoyment and surprise) and negative (sadness and guilt) affective spill-over was observed from competition-extraneous to competition-related concerns, the reverse held true only for disgust. These findings may be due to the athletes' ability to regulate affective reactions within a sporting setting, in particular. Spill-over from competition-extraneous to competition-related concerns is indicative of a lesser degree of control over work/study and family life. Given that average weekly negative affects and anger/disgust were independent predictors of post-competition performance appraisals, the phenomenon of spill-over and other affective linkage mechanisms in sport warrant further investigation.

Empirical evidence suggests that affect, a generic concept including emotions, mood and feelings (Vallerand & Blanchard, 2000), can impact on athletic performance (Hanin, 2000), and that athletic performance can impact on athletes' affective states (e.g., Jones & Sheffield, 2007). Although the affect-performance relationships appear to vary across individuals and types of sport (Cerin et al., 2000; Hanin, 2000; Robazza et al., 2006), it is generally maintained that positive affects such as interest, excitement and vigor are associated, or perceived to be associated, with better performance (Hanin, 2000). Negative affects typified by disengagement behavior and non-task-related rumination (e.g., sadness, guilt and shyness) are claimed to be detrimental to performance (Hanin, 2000; Lane & Terry, 2000; Cerin, 2003). Anger and other hostility-related emotions have been reported to be potentially facilitative to performance in contact sports (Terry & Slade, 1995; Ruiz & Hanin, 2004b; Robazza et al., 2006). These findings suggest that appropriate emotion regulation practices may help athletes optimize their performance. To assist emotion regulation, it is important to identify potential determinants of pre-competition affects that practitioners need to consider.

A considerable number of studies have focused on personal and situational determinants of pre-competition and competition-related affects (e.g., Cerin et al., 2000; Hanin, 2000; Hanton et al., 2003; Cerin, 2004; Nicholls et al., 2009a, b). Here, by pre-competition affects we refer to the affective states experienced in the period leading to a competition irrespective of their cause, whereas by competition-related affects we refer to states that reflect an athlete's appraisal of the competition. To the authors' knowledge, no attempt has been made to determine the extent to which positive and negative events, situations or cognitions from domains other than sport/competition influence how athletes feel about a forthcoming contest, although non-performance-related factors, such as personal problems and study-related concerns, have been identified as barriers to optimal performance states (Ruiz & Hanin, 2004b). It is also unclear whether and how events, situations and cognitions associated with a forthcoming competition may influence athletes’ emotional reactions to events occurring in other domains. Quantitative and in-depth qualitative studies have shown that elite athletes experience stress from both competition-related and competition-extraneous sources (Gould et al., 1993; Nicholls et al., 2009a, b). For instance, lack of finances, worry about school, life-career concerns, substance abuse and family problems are only few of the competition-extraneous stressors that were observed in a group of figure skaters (Gould et al., 1993). Nicholls et al. (2009a) reported elevated sources of life stress in professional rugby players for the domains of diet, climate, sleep and health. Given that affective states can impact on performance and general psychological well-being, it would be pertinent to quantify the eventual effect of competition-extraneous concerns on competitive affects and competition-related concerns on competition-extraneous affects. This type of information is important for planning psychological interventions aimed at performance and well-being enhancement.

## Defining competition-related and competition-extraneous concerns

By “concerns”, we refer to events, situations or cognitions to which athletes attribute their current affective states. They represent dispositions to desire the occurrence or non-occurrence of a given type of event or situation (Frijda, 1986). Therefore, concerns can be personally desirable (goal congruent) or undesirable (goal incongruent). Here, competition-related concerns are defined as those explicitly associated with a forthcoming competition or preparation for a competition, including (1) consequences of practice sessions (e.g., injuries, good performance, interpersonal relationships with teammates or coach); (2) thoughts about the expected performance (e.g., lack of perceived readiness, worries about opponents) and (3) thoughts about expected physical (e.g., suitability of competitive venue for warm-up) or social environmental factors (e.g., biased umpires, social support or pressure from teammates and coach) at the competition. These have been identified previously as salient competition-related stressors (e.g., Gould et al., 1993; Nicholls et al., 2009b) and barriers to optimal performance states (Ruiz & Hanin, 2004a).

Competition-extraneous concerns are those originating from domains other than competitive sport. These include study (formal education), family and home (e.g., household activities, parenting, caring for family members), social network (friends), travel (e.g., commuting to and from work), work, recreation (passive and active pursuits other than an athlete's sport), climate, self-care (e.g., hygiene, diet and sleep) and health (e.g., suffering from a cold) (Nicholls et al., 2009a). Some of these domains are not mutually exclusive as, for example, there may be some overlap between work and social network (friendship between colleagues).

## Defining mechanisms linking affective reactions to competition-related and competition-extraneous concerns

Linking mechanisms seek to explain how two conceptually distinct domains influence each other (Edwards & Rothbard, 2000). They have been a focal topic in the field of organizational psychology for decades where they have been used to explain relationships between family- and work-related affect, values, skills and overt behaviors (Roehling et al., 2003). This study focuses on linkages between affective states arising from concern, rather than activity and domains. The advantage of studying concern domains is that they are more encompassing than their activity counterparts as they also include phenomena (i.e., health and weather conditions) that do not fall under the realm of activities but can potentially impact on competition-related affects. Furthermore, this approach facilitates differentiation between apparent and proper between-domain affective relationships. In fact, it is possible to experience competition-related concerns and affects in a work context, as well as it is possible to experience work-related concerns and affects while preparing for a competition. These situations, by themselves, do not entail relationships between competition- and work-related constructs (Edwards & Rothbard, 2000). Rather, they describe experiences transferred intact between domains. By studying relationships between concern domains, we can ensure the classification of affective states into the correct domain.

Four mechanisms are used to explain the linkage between affective states generated in two different domains: spill-over, segmentation, compensation and congruence (Edwards & Rothbard, 2000). Affective *spill-over* is defined here as the effect of two concern domains (in this study, competition-related and competition-extraneous) on one another that generates between-domain similarities in emotions [defined as sudden, short-lasting reactions to a specific, identifiable actual or imagined event (i.e., concern) leading to physiological and experimental changes and object-focused behavior; Vallerand & Blanchard (2000)]. Affective spill-over across competitive and other domains can be quantified by examining the associations between emotional reactions evoked by concerns in one domain and those arising from temporally adjacent concerns in another domain. Significant relationships would indicate that affects caused by competition-extraneous events or cognitions impinge on competition-related affects or vice versa.

Affective *segmentation* refers to the separation of competition-related and competition-extraneous concerns, such that the two domains do not affect one another. Nil associations between emotions experienced in the two domains would support the segmentation model and indicate that athletes compartmentalize their competitive and non-competitive activities and experiences so that affective reactions and stresses from one domain remain independent from other domains. Affective *compensation* is manifested in efforts to offset dissatisfaction in one domain by seeking satisfaction in another domain. Negative associations between competition-related and competition-extraneous emotions would be supportive of a compensation model. Affective *congruence* is analogous to affective spill-over in that it refers to similarities in affects between two domains. However, while spill-over attributes these similarities to the effect of one domain on the other, congruence attributes the similarities to a third variable affecting both domains (e.g., personality traits, behavioral styles, social norms). The differentiation between spill-over and congruence can be facilitated by a comparison of putative spill-over or congruence effects with carry-over effects, defined here as the relationships of domain-specific emotional reactions with subsequent domain-unspecific moods [relatively long lasting, diffuse, affective state that has no apparent triggering stimulus (Vallerand & Blanchard, 2000)]. The greater the similarity between carry-over and potential spill-over/-congruence effects, the higher the likelihood that the latter are congruence effects (e.g., due to personality traits).

Mechanisms linking sport/competition and other life and concern domains have not been studied per se. However, their existence is implicitly assumed by the Individual Zone of Optimal Functioning (IZOF) model (Hanin, 2000, 2003, 2007) and the interactional model of competitive stress (Cerin et al., 2000). Both models postulate that the athletes' sport-related affective states change across time partly due to the influence of changing context or activity settings. The IZOF model also adds form, content and intensity as dimensions relevant to the study of emotion dynamics (Hanin, 2003). Similarities (possible spillover effects) and dissimilarities (possible segmentation and compensation effects) between affects while shifting from pre-game to mid-game and post-game situations (Hanin, 2003, 2007) and between different types of training and competitive situations (Hanin & Syrjä, 1997) have been reported. However, no study has to date examined the linkages between competition-related and competition-extraneous settings.

## Practical meaning of linkages between concern domains

Work/family research distinguishes negative and positive spill-over effects (Edwards & Rothbard, 2000). Negative spill-over occurs when problems and stresses in one domain drain and preoccupy an individual, thus exhibiting a negative influence on his/her behavior and experiences in another domain. Positive spill-over occurs when satisfaction and stimulation in one domain translate into higher levels of energy and satisfaction in another domain. While this classification may be pertinent to the general well-being, it does not suit the domain of competitive sport where performance is a key issue. As noted earlier, affect valence does not coincide with affect functionality (Cerin, 2004; Ruiz & Hanin, 2004b; Hanin, 2007). In this respect, the IZOF model distinguishes negative optimal, negative dysfunctional, positive optimal and positive dysfunctional affects. In the context of competitive sport, it makes more sense to classify spill-over and other affective linkage effects according to the IZOF model as it considers effects on athletic performance as well as well-being.

## A method for studying affective linkages between concern domains

Mechanisms linking affective states across domains are best studied using daily-process study designs such as the Experience Sampling Method (ESM), whereby sources of concerns and affective states are repeatedly assessed over multiple days in the participants' habitual environment (Alliger & Williams, 1993; Hormuth, 1986). Typically, participants carry small devices (e.g., beepers or pre-programmed watches) signaling the time when they need to complete a questionnaire. The signals are randomly scheduled to account for expectancy effects. This approach can give a detailed picture of how affects and cognitions change in response to naturally occurring cognitions and events. It allows an examination of affective reactions to events/cognitions as well as the extent to which affective reactions linger into subsequent assessment periods. This type of design minimizes the negative effects of retrospective recall biases and allows an examination of intra-individual associations and inter-individual differences in these associations (Hormuth, 1986).

## Aims and hypotheses

This paper presents findings from a broader ESM project aimed to provide a detailed process analysis of athletes' affective states, stressful events and cognitive appraisals during the week leading to, and 3 days after, a major competition (see Cerin & Barnett, 2006, in press). The specific aims of the project were to examine (1) temporal patterns of pre-and post-competition affects and sources of concerns (Cerin & Barnett, 2006); (2) personality and cognitive correlates of, and their interactive effects on pre- and post-competition affects (Cerin & Barnett, in press) and (3) the affective linkages between competition-related and competition-extraneous concerns (this paper). Given the relatively small number of assessments and low prevalence of competition-related concerns post-competition (see Cerin & Barnett, 2006), this particular paper is limited to the pre-competition period only.

The main aim of this study was to analyze affective linkages between competition-related and competition-extraneous concerns. As the literature in organizational psychology suggests that affective spill-over across activity and concern domains is far more common than compensation and segmentation, especially in individuals with a reasonable level of satisfaction in their career (Roehling et al., 2003), we hypothesized that, during the study period, athletes would experience affective spill-over from competition-related to competition-extraneous concerns and vice versa.

A secondary aim of this study was to examine the extent to which the average overall, competition-related and competition-extraneous affective states experienced in the week preceding a contest explained post-competition performance appraisals over and above self-reported performance expectations. Independent effects of pre-competition affects on athletes' appraisal of their actual performance would provide some support for a causal relationship between affective states and performance. Importantly, independent effects of competition-extraneous affects would provide support for the conjecture that daily competition-extraneous concerns may spillover into the competition domain and impair or facilitate task-focused behavior and energy utilization during the competition. This would be especially true if no affective spill-over was observed from competition-related to competition-extraneous concerns.

## Methods

### Participants

Tae Kwon Do and Karate practitioners from major British clubs who were planning to take part in the national championships were approached in person or by telephone and briefed about the aims of the study. Forty-four black-belt, male Tae Kwon Do (*n* = 22) and Karate practitioners (*n* = 22) agreed to participate (response rate 69%). For 38 athletes, this was the major event in the competitive season, while the other six participants also competed at the international level. Thirty-nine out of 44 participants completed the study. Two participants dropped out within 72 h due to believing that the study procedure was too demanding, while three participants discontinued participation due to injuries or other health problems.

Participants ranged in age from 16 to 53 years (overall: 26.77 ±7.75; Tae Kwon Do: 27.00 ± 6.16; Karate: 26.53 ± 9.53). Approximately 50% of them fell into the 21–30 age bracket. The remainder was equally distributed between the youngest ( ≤ 20 years) and oldest ( ≥ 31 years) age groups. They had a mean training experience of 10.40 years (SD = 4.47; Tae Kwon Do: 9.45 ± 4.05; Karate: 11.65 ±4.15). When compared with the norms for male American adults (Costa & McCrae, 1992), this group of athletes exhibited average neuroticism (52nd percentile; Tae Kwon Do: 77.40 ± 18.05; Karate: 73.26 ± 22.45) and above average extraversion (75th percentile; Tae Kwon Do: 120.35 ± 15.76; Karate: 119.84 ± 17.12) as measured by the Revised NEO Personality Inventory (NEO PI-R), Form S (Costa & McCrae, 1992). The sample had a mean level of competitive trait anxiety, as measured by the Sport Competition Anxiety Test (SCAT)-Form A, corresponding to the 60th percentile of the norms for male wrestlers (Martens et al., 1990) (Tae Kwon Do: 23.75 ± 2.95; Karate: 21.21 ± 4.42).

### Materials

#### Person-level information

Demographic information was obtained through a short questionnaire assessing age, training experience, level of participation and perceived current performance. Competition performance expectations were measured on a 11-point Likert scale ranging from 0 (*very much below my usual standard*) to 10 (*very much above my usual standard*) at the beginning of the study and 1 h before the competition. A similar item was used to measure actual performance appraisals immediately after the contest.

The SCAT, Form A was used to measure competitive trait anxiety (Martens et al., 1990). Neuroticism and extraversion were assessed using the NEO PI-R, Form S (Costa & McCrae, 1992). These personality questionnaires are not relevant to the present paper and, thus, their metric characteristics and purpose are described in greater detail in the companion publications (Cerin & Barnett, 2006, in press). Notably, one of these publications examined the independent and moderating effects of sport-related and generic personality traits on pre- and post-competition affects (Cerin & Barnett, in press).

#### Event-level information

Participants were given a booklet containing questionnaires assessing affective states and sources of concerns (events or cognitions). Each booklet included enough experience sampling questionnaires to last for the entire period of sampling. To deliver the random signals for questionnaire completion at five different times a day for 7 consecutive days, Motorola (model: PageOne Minicall) pagers were used. Calls were performed by means of a personal computer and a modem using the AvantPager 32 (version 4.00) software.

The Differential Emotions Scale-IV (DES-IV; Izard, 1991) was used to assess affective states (i.e., emotions and moods). It is a self-report instrument designed for the use and assessment of an individual's experience of fundamental emotions as conceptualized by the differential emotions theory (Izard, 1991). The DES-IV comprises 12 three-item subscales gauging the emotions of interest, enjoyment, surprise, sadness, anger, disgust, contempt, fear, guilt, shame, shyness and self-hostility. The instructional set used in this study was “Read each statement and … indicate how you feel right now.” The answers are given on a 5-point Likert-like scale ranging from *not at all* to *very much so*. Possible intensity scores on each subscale range from 3 to 15. In previous studies, internal consistencies of the individual scales ranged from 0.60 (Shame scale) to 0.85 (Sadness and Anger scales) (Izard et al., 1993). Given that there are only three items in a subscale, these coefficients represent acceptable levels of internal consistency. Several studies have provided support for the construct validity of the DES-IV, including evidence on factorial integrity (e.g., Izard et al., 1993) and criterion validity (e.g., Carey et al., 1997). In the present study, 11 of the 12 emotion scales exhibited an adequate degree of internal consistency (0.73–0.96). Average Cronbach's a for the contempt scale was below 0.45. Consequently, it was excluded from subsequent data analysis.

Similarly to previous ESM studies on daily stress (e.g., van Eck et al., 1998), sources of concern were assessed by asking the participants to describe a positive or negative event, situation or thought (if any) that occurred in the interval since their last self-report and affected their current emotional state. The participants also rated the desirability of the reported concerns from a personal goal perspective. Desirability was defined as a dichotomous variable (desirable vs undesirable). The reported sources of concern were coded according to the activity context with the categories competition-extraneous and competition-related. These categories were mutually exclusive. Only sources of concern for which it was explicitly stated that they were associated with the forthcoming competition were classified as competition-related. For this paper, inter-rater agreement between two independent coders was assessed for 519 events (pre-competition week) using Cohen's κ. Cohen' κ was 0.98 for competition-related concerns and 0.99 for competition-extraneous concerns.

### Procedure

The study was approved by the ethics committee of the local university (Nottingham, UK). During an initial interview, participants were briefed about the aims and procedures of the study, and informed consent was obtained. Anonymity and confidentiality of responses were assured. Participants then completed a demographic questionnaire, the SCAT, expected performance item and the Neuroticism and Extra-version scales of the NEO PI-R. Participants were given a pager, and a booklet containing multiple copies of the DES-IV and items assessing sources of concern to last for the entire period of sampling. They went through a practice session to familiarize themselves with the study protocol.

Participants were paged five random times a day over a period of seven consecutive days before the competition. The day was divided into five blocks between the hours of 09:00 and 21:30 hours. Within each of these periods, one randomized pager signal was sent with a minimum of 30 min delay between the signals. Upon reception of the signal, participants completed an experience sampling questionnaire. They first indicated the date and time of the day of completion. Second, they rated their momentary affective states on the DES-IV. Finally, they reported an eventual positive or negative source of concern (if any) experienced in the interval since their last report. During the data collection, athletes were not explicitly asked competition-related questions to avoid diverting their attention to the forthcoming contest and to examine the natural flow of affects and perceived sources of concern (events or cognitions) in their habitual environment. Participants were instructed that if the pager was accidentally turned off or malfunctioned, or if they were unable to answer within 30 min of the signal, they should not complete the questionnaires for that sampling. On the day of the competition, the participants completed the usual set of questionnaires approximately 1 h before the competition. Following the standard recommendations for ESM studies and in order to minimize attrition and selection bias due to the intrusiveness of the study protocol, an inconvenience allowance of £35 was given to the participants who completed the study (Hormuth, 1986; Christensen et al., 2003).

During the week preceding the competition, participants completed an average of 94% of all possible responses within the time limit, for an average of 32.9 out of 35 valid responses per participant. The average time delay between the signal from the pager and the reported time of completion of the questionnaires was 7.88 min (SD = 8.49). Compliance rate was unrelated to demographic characteristics, personality traits and day of the study.

### Data manipulation and analysis

Between-day, between-subject and within-subject standard deviations and means of affects associated with competition-related, competition-extraneous and no concerns were computed. Concern transitions as occurring when participants reported changes in the presence and type of concern across adjacent ESM assessments within the same day were identified to examine affective linkages. As previous research suggests that, for the average individual, affective reactions to daily events tend not to carry over into subsequent days (Bolger et al., 1989), transitions between the last assessment of a day and the first assessment of the following day were not examined. Four types of transition were identified. These were: (1) competition-extraneous to competition-related concerns; (2) competition-related to competition-extraneous concerns; (3) competition-extraneous to no concern and (4) competition-related to no concern.

Separate multilevel linear models with random intercepts, but fixed regression coefficients, were estimated for each of the four types of transition for each affective state. Regression coefficients were not allowed to vary across days and subjects due to the small daily average number of concern transitions per subject (0.3–0.8). Multilevel linear models are a variant of the multiple regression models, which is appropriate for datasets with a multilevel (hierarchical) structure (Snijders & Bosker, 1999). They are particularly useful for the analysis of longitudinal data, allowing for missing observations and observations unequally spaced in time.

As, in this study, the dataset comprised of one or more daily observations on affects nested within days within subjects, the models included three levels of variations. These were concern-transition level (variations in the outcome across concern-transition events within a day within a person), day level (variations across days within a person) and person level (variations between persons). In step 1 of the analyses, current levels of affect (time = 0) were entered in a model without predictors and variance components of affect were estimated. In step 2, levels of affect from the assessment before the current (time-1) were added to the model. The regression coefficient for affects experienced on the assessment preceding the current (time-1) represented the magnitude of the effect of the affect triggered by a certain type of concern on subsequent affects. Higher order lags were not entered in the model because a “time-1” lag usually accounts for most of the lagged variance in affective states (Alliger & Williams, 1993). Statistical significance of the regression coefficients was established by dividing the estimated effect by its standard error. This ratio is approximately normally distributed (Snijders & Bosker, 1999). Two-tailed tests and a probability level of 0.05 were used. The amount of variance in the current levels of affect explained by a linkage mechanism (i.e., spill-over or compensation) was established by calculating the change in the explained portion of the criterion variance (Δ*R*^2^) after inclusion of affect at time-1 using the method described by Snijders & Bosker (1999) (change in variance from step 1 to step 2 of the models). The regression assumptions of normality, linearity and homoscedasticity were examined using plots of standardized residuals.

To examine whether average pre-competition affective states (general, competition-related and competition-extraneous) experienced during the week leading to the competition explained performance appraisals immediately after the competition, over and above performance expectations, hierarchical regression analyses were performed. Performance expectations were assigned first entry and affects were assigned second entry. To address multicollinearity problems (e.g., positive affects tend to be moderately to highly correlated) and the small sample size (*N* = 39), average ratings for negative affects (shyness, shame, sadness, guilt, fear and self-hostility), positive affects (surprise, interest and enjoyment) and anger/disgust were computed and entered as predictors in the regression models. These groups of affects were determined via principal components analyses of the subject-aggregated mean scores and within-subject z-scores on the DES-IV subscales (results available on request). These three factors accounted for 67.7% and 64.5% of the total between-subject and within-subject item variance, respectively.

## Results

### Descriptive statistics

Eighty-two observations with missing data on any of the predictors were deleted. This resulted in a total of 1283 valid observations. In the week leading to the competition (excluding the day of the competition), athletes reported a total of 190 competition-related and 329 competition-extraneous concerns (see Table 1). This corresponded to 16.6% and 28.8% of the total number of valid ESM reports. Competition-related concerns encompassed thoughts, expectations and conversations about the forthcoming event; performance at training sessions; and injury incurred during training. Competition-extraneous concerns included events and cognitions related to education, work, family, social network, recreation health and travel sub-domains (Table 1). Most competition-related concerns were in the form of desirable thoughts about the forthcoming contest and satisfaction with performance at training. “Making mistakes during training” was the most prevalent category of undesirable competition-related concerns. The most frequently reported desirable competition-extraneous concerns fell within the sub-domains of recreation, family/home and social networks. Work and family/home were the most prevalent sources of negative competition-extraneous concerns (Table 1).

**Table 1:** Sub-domain, content and frequency of competition-related and competition-extraneous concerns

Concern sub-domain and content	*f*	*f*_tc_	*f*_tnc_	Concern sub-domain and content	*f*	*f*_tc_	*f*_tnc_
Desirable competition-related concerns *Forthcoming competition*	166	27	72	Undesirable competition-related concerns *Forthcoming competition*	24	13	8
Thinking about the competition	85	18	60	Worrying about the competition	2	2	0
Talking about the competition	38	4	5	Not feeling ready for the competition	2	1	1
*Training sessions (preparation for competition)*				*Training sessions (preparation for competition)*			
Satisfied with performance	40	4	5	Unsatisfactory performance at sparring	4	1	1
Good coaching	3	1	2	Making mistakes	9	6	3
				Injury	3	3	0
				Unable to focus	3	0	3
				Late for training	1	0	0
Desirable competition-extraneous concerns *Education*	145	26	105	Undesirable competition-extraneous concerns *Education*	184	27	111
Finishing coursework	8	2	5	Problems with coursework	7	2	3
Good performance	4	1	2	Interpersonal problems with teachers	4	0	2
				Difficulties with assessment	4	1	2
				Poor performance	5	0	2
*Family and home*				*Family and home*			
Playing/spending time with child	14	2	10	Unable to see child (divorced)	5	1	4
Having meals with family	6	0	6	Financial difficulties	5	2	3
Working in the garden/yard	3	0	1	Arguments with family members	12	4	4
Planning family holidays	2	0	2	Fixing broken appliances	6	0	4
Relaxing at home with family	16	3	12	Unwanted family commitments	15	1	9
				Family members arguing	13	0	8
*Work*				*Work*			
Accomplished important task	11	3	7	Having to fire someone	5	0	4
Satisfied with job	8	0	4	Discontent among co-workers	11	0	7
Getting pay raise	1	0	1	Heavy work load	20	1	15
				Lack of organization/poor work practice	9	1	7
				Others' misconduct at work	6	2	4
				Making mistakes	7	2	5
				Late for work	3	0	3
				Argument with colleague	8	0	4
*Social network (friends)*				*Social network (friends)*			
Socializing with friends	29	7	20	Disappointment	2	1	0
Helping out friend	6	1	5	Argument with friend	3	1	2
				Disturbed by friends/neighbors	8	0	5
				Friend's illness	1	0	1
*Other sub-domains*				*Other sub-domains*			
Recreation				Health			
Going to the cinema/theatre/concert	6	1	5	Injured at work or home	5	2	2
Hobby	10	4	6	Catching a cold	1	1	0
Playing other sports (soccer, snooker, etc.)	21	2	19	Tiredness	8	2	4
				Overeating	2	1	1
				Hangover	2	1	0
				Travel			
				Near accident	3	0	3
				Heavy traffic	2	1	1
				Vehicle break-down	2	0	2

*f*, frequency; *f*_tc_, frequency with which a type of concern was followed by a concern of different domain; *f*_tnc_, frequency with which a type of concern was followed by no concern.

Levels of negative affects were generally lower than those of positive affects (Table 2). The average ratings on positive affects were 6.25 (SD = 1.92), whereas those on negative affects and anger/disgust were 3.26 (SD = 0.38) and 3.37 (SD = 0.38), respectively. Compared with competition-extraneous concerns, competition-related concerns were associated with higher levels of positive affects and fear, but lower levels of other negative affects. When compared with concern-free occasions, competition-extraneous concerns tended to be accompanied by increases in negative affects, especially anger, and an increase in surprise and interest. Substantial inter-individual and intra-day variations in mean affects across types of concern were observed (Table 2; see person- and inter-day level SD). Fear resulting from competition-related concerns, and anger, enjoyment and surprise resulting from competition-extraneous concerns, were the affects with the largest degree of variation across days of experience sampling (Table 2).

**Table 2:** Mean affects and between-subject, between-day and within-day variability by type of concern

Affect	Competition-related concern (*n* = 190)				Competition-extraneous concern (*n* = 329)				No concern (*n* = 764)			
			
	*M*	SD_bs_	SD_bd_	SD_wd_	*M*	SD_bs_	SD_bd_	SD_wd_	*M*	SD_bs_	SD_bd_	SD_wd_
Guilt	3.35	0.55	0.400	0.77	3.52	0.55	0.19	1.04	3.16	0.40	0.04	0.54
Shyness	3.28	0.43	0.08	0.64	30.3	0.430	0.13	0.68	3.13	0.38	0.00	0.51
Disgust	3.06	0.19	0.00	0.25	0.0	0.00	0.05	1.01	3.09	0.0	0.00	0.54
Self-hostility	3.23	0.33	0.09	0.62	3.40	0.55	0.08	0.94	3.06	0.18	0.03	0.30
Shame	3.34	0.76	0.04	0.54	3.29	0.45	0.040	0.56	3.14	0.45	0.04	0.51
Sadness	3.25	0.46	0.64	0.56	3.65	0.65	0.13	1.10	3.22	0.48	0.00	0.64
Fear	3.84	0.85	0.39	1.08	3.39	0.47	0.00	0.85	3.17	0.35	0.00	0.52
Anger	3.53	0.62	0.19	0.78	4.45	0.83	0.24	2.02	3.26	0.54	0.00	0.70
Enjoyment	8.45	1.90	0.07	2.01	7.02	0.072	0.32	2.40	7.33	2.00	0.29	0.17
Surprise	5.20	1.21	0.0	1.39	4.81	1.32	0.131	1.0	1.01	1.01	0.13	1.08
Interest	8.02	1.72	0.0	5.86	6.30	1.36	0.10	2.27	5.86	1.60	0.32	1.76

SD_bs_, between-subject level standard deviation; SD_bd_, between-day level standard deviation; SD_wd_, within-day level standard deviation; *n*, number of reported concerns

On 67 occasions, a specific concern was followed by a concern from the same domain (52 competition-related and 15 competition-extraneous concerns). On 63 instances, a concern was reported at the first assessment of the day (18 competition-related and 45 competition-extraneous concerns). Affective states reported on these assessments were not examined in the regression models of affective linkages because they do not represent concern transitions.

The most frequently experienced type of concern transition was from competition-extraneous to no concerns (Table 3), whereas the least frequently experienced was from competition-related to competition-extraneous concerns. In the week leading to the competition, all participants reported at least one transition from a competition-related or a competition-extraneous concern to no concern (Table 3). Just over half of the participants reported at least one transition from a competition-related to a competition-extraneous concern. Thoughts about the forthcoming event were the desirable competition-related concern, while making mistakes at training were the undesirable competition-related concern most frequently followed by no concern or a competition-extraneous concern. Desirable competition-extraneous concerns falling within the sub-domains of family/home, work, social networks and recreation were most frequently followed by no concerns or competition-related concerns. For undesirable competition-extraneous concerns, these were concerns related to the sub-domains of family /home and work.

**Table 3:** Affective spill-over across competition-related (CRC), competition-extraneous (CEC), and no concerns: results of multilevel regression analyses

Affects	CEC to CRC (*n*_t_ = 53; *n*_p_ = 24)		CRC to CEC (*n*_t_ = 40; *n*_p_ = 20)		CEC to no concern (*n*_t_ = 216; *n*_p_ = 39)		CRC to no concern (*n*_t_ = 80; *n*_p_ = 39)	
				
	*b* (SE)	Δ*R*^2^	*b* (SE)	Δ*R*^2^	*b* (SE)	Δ*R*^2^	*b* (SE)	Δ*R*^2^
Guilt	0.47 (0.13)***	0.23	0.16 (0.37)	0.01	0.35 (0.07)***	0.12	0.34 (0.07)***	0.19
Shyness	−0.23 (0.16)	0.01	0.36 (0.25)	0.02	0.47 (0.08)***	0.16	0.23 (0.06)***	0.23
Self-hostility	0.09 (0.15)	<0.01	0.24 (0.70)	<0.01	0.13 (0.04)***	0.09	0.71 (0.04)***	0.75
Shame	0.02 (0.23)	<0.01	0.09 (0.16)	0.01	0.26 (0.12)*	0.08	0.11 (0.05)*	0.09
Sadness	0.37 (0.05)***	0.56	0.16 (0.23)	<0.01	0.29 (0.06)***	0.14	0.69 (0.10)***	0.28
Fear	0.31 (0.18)	0.02	0.25 (0.24)	0.01	0.42 (0.06)***	0.20	0.34 (0.07)***	0.28
Disgust	0.03 (0.12)	<0.01	0.43 (0.13)***	0.06	0.29 (0.09)***	0.06	0.51 (0.12)***	0.23
Anger	0.01 (0.04)	<0.01	−0.03 (0.35)	0.01	0.18 (0.03)***	0.12	0.54 (0.10)***	0.32
Enjoy	0.36 (0.10)***	0.26	0.260 (0.22)	0.02	0.32 (0.05)***	0.20	0.38 (0.09)***	0.19
Surprise	0.30 (0.10)**	0.20	0.15 (0.24)	0.01	0.13 (0.05)**	0.07	0.43 (0.09)***	0.24
Interest	0.08 (0.16)	0.1	0.18 (0.14)	0.02	0.18 (0.05)***	0.08	0.23 (0.09)**	0.12

*Note*: The unstandardized regression coefficients (*b*) represent the effect of affective states arising from competition-related or competition-extraneous concerns at time-1 on current affective state. Δ*R*^2^ represent the proportion of the criterion variance explained by affects at time-1.

* *P*<0.05

** *P*<0.01

*** *P*<0.001.

CEC, competition-extraneous concerns; CRC, competition-related concern; SE, standard error of regression coefficient; *n*_t_, number of concern transitions; *n*_p_, number of participants experiencing a type of transition.

Mean performance expectations at the start of the study and 1 h before the contest were 6.18 (SD = 1.54) and 6.07 (SD = 1.64), while performance appraisal was 5.46 (SD = 1.64). This indicates that athletes, on average, appraised their performance to be slightly lower than what they, on average, had expected.

### Affective linkages

In general, the data did not provide sufficient evidence for affective spill-over from competition-related to competition-extraneous concerns (Table 3). A significant effect was observed for disgust. An examination of the regression coefficients indicated, that in some cases, the lack of a significant spill-over effect might have been due to the small number of transitions falling into this category (lack of power). Significant spill-over from competition-extraneous to competition-related concerns was observed for guilt, sadness, enjoyment and surprise, whereas the estimated effects for self-hostility, interest, shame, disgust, and anger approached zero suggesting some segmentation. No evidence of affective compensation was found. Affects associated with competition-related and competition-extraneous concerns persisted (at least) until the following concern-free assessment, with competition-related concerns showing a stronger effect than competition-extraneous concerns.

Affects experienced in association with a competition-extraneous concern explained from 6% to 20% and those associated with a competition-related concern from 9% to 75% of the affect variance in the subsequent concern-free assessment.

### Pre-competition affect and competition performance appraisals

After accounting for performance expectations, the mean weekly level of overall anger/disgust (irrespective of the type of concern reported), was positively, whereas negative affects were negatively related to actual performance appraisals (Table 4). In all regression models, performance expectations were positively associated with actual performance appraisals. Positive affects arising from competition-related concerns were significantly positively, whereas competition-extraneous negative affects were significantly negatively, associated with performance appraisals.

**Table 4:** Independent associations of average pre-competition affects and appraisals of performance at the competition: results of hierarchical regression analyses

Predictors	*b* (SE) All ESM reports (*n* = 1283)	Competition-related concerns (*n* = 190)	Competition-extraneous concerns (*n* = 329)
*Performance expectations*#	0.55 (0.21)*	0.58 (0.21)**	0.49 (0.22)*
Affect			
Positive (enjoyment, interest, surprise)	0.08 (0.18)	0.46 (0.20)*	0.30 (0.23)
Negative (guilt, shyness, shame, self-hostility, sadness, fear)	−4.63 (1.67)**	−1.92 (1.17)	−1.73 (0.83)*
Anger/disgust	3.45 (1.61)*	1.41 (1.50)	0.81 (0.53)
Δ*R*^2^	0.17	0.18	0.13

*Note*:

# Average performance expectations at the start of the study and 1 h before the contest.

* *P*<0.05

** *P*<0.01.

*b*, unstandardized regression coefficients; SE, standard error of regression coefficient; Δ*R*^2^, represent the proportion of the criterion variance explained by affects over and above performance expectations; ESM, experience sampling method.

## Discussion

The primary aim of this study was to examine the mechanisms of affective linkages between competition-related and competition-extraneous concerns. The results provided support for carry-over and spill-over effects, especially from competition-extraneous to competition-related concerns. At the same time, some evidence was found for affective segmentation in the form of absence of relationships between domain-specific affects, while no support was found for compensation effects across concern domains.

A secondary aim of the study was to examine the extent to which overall and context-specific average affective states experienced in the week leading to a competition would explain athletes' performance appraisals after accounting for performance expectations. It was hoped that these findings would provide some insight into the significance and practical implications of eventual spill-over, compensation or carry-over effects with respect to athletic performance. Context-specific and overall pre-competition affects were found to be independently related to performance appraisals confirming the importance of examining mechanisms linking affective reactions to different concern domains. These findings are discussed below.

### Affective carry-over effects

#### Effects of competition-extraneous concerns

Overall, the findings supported the contention that competition-extraneous concerns impact on general pre-competition as well as specific competition-related affects. All positive and negative affects triggered by competition-extraneous concerns tended to linger into the next assessment period. Main sources of concerns included the sub-domains of family/home, social networks, work and recreation. While family/home and work sub-domains were associated with reports of both desirable and undesirable concerns, social networks and recreation were, in the main, sources of desirable concerns.

Considering the fact that ESM assessments were, on average, 2.4 h apart, these carry-over effects appear to have been of relatively long duration and, hence, potentially disruptive to daily training sessions and preparation for the competition. Even small increases in affects typified by disengagement behavior and non-task focus, such as sadness, guilt and shyness (Izard, 1991; Hanin, 2000), have been found to be detrimental to performance (Hanin, 2000, 2003, 2007; Lane & Terry, 2000; Cerin, 2003). In this respect, our study revealed that general negative affects (guilt, shyness, self-hostility, shame, sadness and fear) were predictive of lower performance appraisals even after accounting for performance expectations. Similarly, a sample of high-level karate practitioners identified sadness and fear as affective states associated with poor performance (Ruiz & Hanin, 2004a). As, in this study, competition-extraneous concerns were the most prevalent type of concern in the week leading to a competition, and approximately half of these were considered negative stressors (Cerin & Barnett, 2006), addressing problems associated with domains other than sport appears to be an important component of athletes' mental preparation for a forthcoming competition.

Our study also suggests that when positive, competition-extraneous events and cognition may act as energizers for the forthcoming competition. In fact, significant carry-over effects were observed for interest/excitement, which typically enhances the ability to process information from the environment and helps sustain focus on the task (Izard, 1991). However, some of the positive carry-over effects might have been dysfunctional. For example, enjoyment may impair performance if it leads to a decrease in effort and disengagement from the task (Hanin, 2000, 2003, 2007). These potentially mixed effects on performance might explain the lack of significant associations between positive affects and performance appraisals observed in this study.

As noted in the introduction, negative affects, such as anger, are sometimes optimal for performance in martial arts (Terry & Slade, 1995; Ruiz & Hanin, 2004b). However, similar to what has been observed for competitive anxiety (Jones & Swain, 1995), it appears that anger functionality depends on the source and interpretation of anger. High-level karate practitioners reported that facilitative anger was related to readiness to perform and energy generation for task execution, whereas debilitative anger was the result of low readiness to perform and perceived lack of resources (Ruiz & Hanin, 2004b). In this respect, the fact that no significant relationship was found between performance appraisal and anger triggered by competition extraneous-concerns may be due to different athletes experiencing different types of anger at different times. It is also possible that, similar to what has been observed for anxiety (Cerin, 2004), anger functionality is partly determined by anger intensity. These are issues that need to be thoroughly explored in future studies.

#### Effects of competition-related concerns

As with competition-extraneous concerns, positive and negative affects triggered by competition-related concerns persisted at least until the next ESM assessment. However, these carry-over effects tended to be stronger, a finding likely to reflect the importance that was attributed to the contest. The largest carryover effects were observed for self-hostility and sadness, indicating that athletes were particularly reactive to competition-related cognitions and events associated with a real or potential failure to attain their competitive goals. In fact, an analysis of the content of undesirable concerns indicated that making mistakes and being unable to focus during training were the likely causes of such negative carry-over effects.

### Affective spill-over, segmentation and compensation effects

#### Competition-extraneous concerns

As hypothesized, some negative and positive affective spill-over were observed from competition-extraneous to competition-related concerns, with guilt and sadness showing the strongest effects. These negative spill-over effects are likely dysfunctional since guilt and sadness are generally associated with deactivation, and submissive and avoidance behavior (Izard, 1991). As such, they are not helpful to performance in sport (Hanin, 2000), and especially in contact sports (Robazza et al., 2006). Importantly, this study found a significant detrimental effect of negative affective states triggered by competition-extraneous concerns on performance appraisals. It is also noteworthy that although in this study most of the competition-related concerns were considered desirable (see Cerin & Barnett, 2006), irrespective of whether they were preceded by a pleasant event or no event, 17 out of a total of 24 undesirable competition-related concerns (71%) were preceded by an undesirable competition-extraneous concern. In contrast, only 6% of positive competition-related concerns followed negative competition-extraneous concerns. These findings point to the presence of a spill-over effect of practical significance, whereby competition-extraneous stressors influenced the way athletes' approached and felt about a forthcoming athletic contest. Although this type of spill-over was relatively infrequent and occurred in only 62% of the participants, it cannot be ignored due to what is already known about the relationship between affects and performance.

Affective spill-over was also found for enjoyment and interest, suggesting that positive competition-extraneous concerns tend to have a positive effect on how athletes psychologically and emotionally react to competition-related concerns. Given that this study found that competition-related positive affects were predictive of performance appraisals, this finding is also of practical importance to athletes and sport psychologists, who need to appreciate the significance of maintaining a reasonable level of satisfaction in life domains other than competitive sport. Investing all time and efforts in one's sport to the detriment of other aspects of life may not be a wise choice with respect to an athlete's well-being as well as athletic performance (Kallus & Kellmann, 2000).

Although some affective spill-over was found, cross-domain segmentation effects were evident for most negative affects and interest, indicating that athletes were capable of compartmentalizing negative competition-extraneous concerns from competitive sport. With regard to interest, it is not unexpected that the level of interest in a wide range of life domains did not parallel that in martial arts.

#### Effects of competition-related concerns

This study did not provide sufficient support for affective spill-over from competition-related to competition-extraneous concerns. A significant effect was observed for disgust only. However, the magnitude of this effect was smaller than the corresponding carryover effect suggesting that the observed association might be the result of a third variable (affective congruence) rather than a genuine manifestation of cross-domain spill-over (Edwards & Rothbard, 2000). Although these non-significant results could be due to the small number of this type of concern transition, it is also possible that the examined samples of athletes were able to control their psychological reactions related to the competition and their sport so that they would not interfere with other life domains. Self-control and emotion regulation are important components of the martial arts (Konzak & Bourdeau, 1984). Several studies have found the long-term practice of martial arts to be associated with increases in self-control (Brown et al., 1995), self-confidence (Spear, 1989) and decreases in hostility and anger (Nosanchuk & MacNeil, 1989; Daniels & Thornton, 1990; Brown et al., 1995). The regulation of anger and hostility is seen as particularly important in martial arts from the stand-point of personal development (Konzak & Bourdeau, 1984) and improved strength performance (Murphy et al., 1988). Proficiency in anger regulation might explain why no sign of spill-over were found for the affects of anger and self-hostility across competition-related and competition-extraneous concerns and vice versa.

The fact that evidence of affective spill-over was found from competition-extraneous to competition activities but not from competition to competition-extraneous activities could be due to athletes having a greater control over the sport/competition sphere of activity than over the family and/or work/education domains. In this respect, studies on work-family spillover have found that the reason why employment has more of a negative impact on family life than family life has on work life is the relative inflexibility of and lower degree of control over decisions in the work life compared with family life (Roehling et al., 2003). Involvement in sport and competition is a free-choice activity usually characterized by high levels of perceived control over participation at least. Consequently, it is unlikely to exert a pronounced negative effect on other life domains. In contrast, unavoidable work/education and family commitments may more often interfere with competitive activities in terms of resources and time allocation. Additionally, the ability to exert emotional control gained as a result of practicing martial arts may be somewhat context specific, which would mean that these athletes can more easily regulate affects generated within a sporting context than those arising in domains where affective control is not perceived as an integral part of the activity (Gross, 2007).

### Practical implications

This study indicates that competition-extraneous concerns can influence the way athletes feel about a forthcoming competition and that emotions triggered by such concerns may potentially influence performance. The presence of such an influence, especially if negative and dysfunctional in nature, would call for the implementation of emotion regulation strategies and counseling targeted towards competition-extraneous “problem” areas (typically, work/education and family/home). Practitioners need to take into consideration both valence (negative or positive) and functionality (optimal or dysfunctional) of a specific affective linkage between competition-extraneous and competition-related concerns. While valence is important for an athlete's general well-being, functionality is important for athletic performance. It is straightforward to identify the valence of an affective linkage as this is defined by the type of affect and relationship between concerns. However, the functionality of an affective linkage is to a large extent idiosyncratic and needs to be assessed individually (Hanin, 2000, 2003, 2007). Although the detrimental effects of affective states that clearly lead to task disengagement (e.g., sadness and guilt) on performance appear to be universal, the functionality of affective states with variable action tendencies (e.g., enjoyment, anxiety, anger) need to be determined on an individual basis and across various contexts (Hanin, 2003, 2007; Ruiz & Hanin, 2004b).

### Limitations and future avenues

Although this study provides a valuable, relatively rare daily-process analysis of affective states and linkage mechanism across concern domains in the week before a competition, it also presents several limitations. First, participants reported only one source of concern per ESM assessment, while they might have sometimes experienced multiple concerns. Secondly, this study did not assess the activity context in which the affective states and concerns were experienced. Namely, participants might have reported competition-related concerns (thoughts) during work or study. To gain a clear idea of the affective linkage phenomena between competition-related and competition-extraneous domains, it would be necessary to know the settings in which the affective states and concerns were experienced. Thirdly, desirability was measured as a dichotomous variable, while a continuous scale would allow the identification of sources of concern most likely to elicit emotions of a specific valence, important to the athletes’ well-being. Fourthly, because of the limited sample size of concern transitions, no attempts were made to identify personal and situational correlates and moderators of cross-domain affective linkages. From an applied standpoint, it would be useful to study between-domain affective linkages using a single-subject paradigm (idiosyncratic approach; Hanin, 2000) and then identify the characteristics underlying individual differences by pooling data from multiple single-subject studies. Fifthly, given that this ESM study was not conducted using personal digital assistants, which automatically provide a time stamp for each ESM assessment, it is possible that participants did not comply with the procedure of the study and provided retrospective rather than real-time information. Sixthly, this study did not objectively assess actual performance but collected information on subjective appraisals of the performance using a single-item scale. Finally, this study collected data on a sample with an extreme age range (16–53 years). Given that life domains and sources of concern change considerably across the life span, it would have been optimal to analyze affective linkage effects by age groups. However, the limited number of participants precluded such detailed analyses. All these weaknesses need to be addressed in future studies.

Mirroring the long tradition of cross-domain affective linkages research in organizational psychology, future investigations will need to clarify the direction and magnitude as well as personal and situational determinants of these phenomena among athletes. These may include gender, type of sport, level of participation, role involvement, social support and structure of a competitive season. Future investigations also need to further clarify the types of competition-extraneous domains (e.g., family and work) and concerns that exert greater effects on competition-related concerns and activities. It is important that research in this field follow both nomothetic and idiographic approaches in determining the functionality of specific affective linkage effects. Finally, studies of cross-domain affective linkages need to be extended post-competition and undertake a more detailed analysis of influences between sub-domains (e.g., work to training; family/home to competition).

## Perspectives

Affective linkages between competition-related and competition-extraneous concerns and domains are a topic worth pursuing in the field of sport psychology. The findings of this study suggest that competition-extraneous events and cognitions may influence athletes' competition-related and pre-competitive affects, which in turn may influence the preparation for and the performance at a competition. Work and family/home domains are likely salient sources of concerns that yield negative or positive affective linkage effects, some of which may be dysfunctional and others optimal. Social networks and recreation are domains that, in the main, elicit positive linkage effects, some of which may be dysfunctional and others optimal. To be practically meaningful, the functionality of affective linkages needs to be established on an individual basis and across various sport contexts (e.g., training and competition). Competition-related concerns may have an impact on athletes' general well-being by triggering affective states that persist in time. Pre-competition, most competition-related concerns are positive and, thus likely to be beneficial to an athlete's well-being. However, when negative, their effect is substantial and non-ignorable. Practitioners are encouraged to identify and monitor affective linkage effects of salient competition-related and competition-extraneous concerns, which may help devise emotion regulation strategies that foster athletes' optimal affective states and well-being.
